# COVID-19 Illness Severity in the Elderly in Relation to Vegetarian and Non-vegetarian Diets: A Single-Center Experience

**DOI:** 10.3389/fnut.2022.837458

**Published:** 2022-04-29

**Authors:** Yi-Cheng Hou, Wen-Lin Su, You-Chen Chao

**Affiliations:** ^1^Department of Nutrition, Taipei Tzu Chi Hospital, Buddhist Tzu Chi Medical Foundation, New Taipei City, Taiwan; ^2^Division of Pulmonary and Critical Care Medicine, Department of Internal Medicine, Taipei Tzu Chi Hospital, Buddhist Tzu Chi Medical Foundation, New Taipei City, Taiwan; ^3^School of Medicine, Tzu-Chi University, Hualien, Taiwan; ^4^Division of Gastroenterology and Hepatology, Taipei Tzu Chi Hospital, Buddhist Tzu Chi Medical Foundation, New Taipei City, Taiwan

**Keywords:** COVID-19, diet, Taiwan, illness severity, nutritional status

## Abstract

The first wave of the coronavirus disease 2019 (COVID-19) outbreak in Taiwan occurred in May 2021. The risk for and severity of this disease vary and are highly dependent on personal habits and comorbidities. Moreover, the gut microbiome, which may be affected by diet, is highly susceptible with regard to the risk and severity of infectious diseases such as COVID-19. The relationship between dietary habits, nutritional status, and the effects of these factors on the immune system in the context of a global pandemic is an extremely important topic of immediate concern. Hence, the aim of this study was to explore the effect of vegetarian and non-vegetarian diets on COVID-19 severity during the pandemic. We conducted a retrospective evaluation of 509 patients who had been diagnosed with COVID-19 at a single medical center between May 2021 and August 2021. Patients were divided into three groups according to disease severity. For patients aged ≥65 years, COVID-19 symptom severity was statistically significantly and inversely associated with the adherence to a vegetarian diet (*p* = 0.013). Moreover, subgroup analysis results showed that older COVID-19 patients and those with a non-vegetarian diet had a higher risk of contracting critically severe COVID-19 [adjusted odds ratio (OR) = 5.434, *p* = 0.005]. Further research is needed to determine the effects of dietary habits on COVID-19 risk and severity during the global pandemic.

## Introduction

In Taiwan, the first confirmed case of coronavirus disease 2019 (COVID-19) occurred on January 21 2020, and the first wave of the COVID-19 pandemic outbreak occurred in May 2021. Community COVID-19 transmission was confirmed as many sources of infection could not be traced. Many locally confirmed cases were identified as uncomplicated or mild COVID-19 according to SARS-CoV-2 RT-PCR (i.e., severe acute respiratory syndrome coronavirus 2 reverse transcription polymerase chain reaction assay) results and clinical symptomology. These confirmed cases were admitted to centralized quarantine centers in accordance with governmental mandates.

Hotels in Northern Taipei were remodeled during the pandemic in order to serve as quarantine centers, and were supported by medical centers. More specifically, these makeshift quarantine centers provided medical care for confirmed cases of asymptomatic to mild COVID-19. If the disease progression of the confirmed cases worsened, these patients were transferred to their respective medical centers for appropriate treatment. Accordingly, there were different levels of COVID-19 severity in this study population (i.e., patients recruited from quarantine centers that were supported by a single medical center), which is hence suitable for research on disease risk factors in the hospital setting.

Dietary influence on the gut microbiome is an extremely important topic overall, as well as in light of susceptibility with regard to the varying risk and severity of infectious diseases, according to nutritional patterns, such as the possibility of healthy plant-based food intake modulating COVID-19 risk (1).

Merino et al. administered web-based surveys targeting healthcare workers with substantial exposure to COVID-19 patients, and analyzed the influence of self-reported dietary patterns on COVID-19 outcomes and severity (2, 3). These researchers found that plant-based diets were associated with a low risk of moderate to severe COVID-19.

The relationship between dietary habits, nutritional status, and the effects of these factors on the immune system in the context of a pandemic is an extremely important topic of immediate concern (3–6). It seems that a healthy lifestyle may reduce the severity of COVID-19 symptomology, and these preliminary findings prompt further investigation (7). Evidence with regard to specific dietary patterns that may support the optimal alleviation of COVID-19 risk and symptomology have not been widely researched. In this study, we aimed to evaluate the association between self-reported dietary patterns and the severity of COVID-19 in a hospital setting.

## Materials and Methods

### Subject Selection

To evaluate the relationship between vegetarian diets and the severity of COVID-19 symptoms, we retrospectively collected data from medical records of patients housed at quarantine centers. Dietary data were queried in questionnaires asking about dietary patterns 1 year before the date of subjects' COVID-19 diagnosis. Data were collected from the corresponding support hospital in Northern Taiwan.

In this cross-sectional study, 509 patients between 20 and 95 years of age, who had been diagnosed with COVID-19 (Roche Cobas® SARS-CoV-2 PCR Reagent; Roche, Basel, Switzerland), were recruited between May 2021 and August 2021. During the pandemic, all 509 COVID-19 patients had not received COVID-19 vaccinations.

Patients were divided into three groups according to disease severity, as shown in Table 1. Patients with incomplete data on the demographic questionnaire and symptom surveys were excluded from the current study.

**Table 1:** Various coronavirus disease 2019 (COVID-19) classification levels.

**Category**	**Laboratory tests**	**Medical care**	**Symptoms**
Uncomplicated to mild COVID-19	Positive SARS-CoV-2 PCR result or a positive antigen test result	Receiving outpatient or inpatient care, but not HFNC oxygen or mechanical ventilation	Patients with mild symptoms and patients with an uncomplicated upper respiratory tract viral infection may present with fever, cough, sore throat, nasal congestion, malaise, headache, or muscle pain
Moderate to severe COVID-19	Laboratory-confirmed SARS-CoV-2 infection	Standard of care: need for urgent hospital treatment	Patients with severe pneumonia and clinical signs of pneumonia (fever, cough, dyspnea, fast breathing) and one or more of the following: • Respiratory rate > 30 breaths/min • Severe respiratory distress • SpO_2_ < 94% on room air • PaO2/FiO2 < 300 • Infiltration > 50%
Critical COVID-19	Presumed or confirmed SARS-CoV-2 infection	Standard of care: ICU admission for respiratory or cardiovascular organ support; need for intubation, rescue strategies, or oxygenation (i.e., achieving a change in the PaO_2_/FiO_2_ ratio)	Presence of acute respiratory distress syndrome, respiratory failure requiring ventilation, sepsis, or septic shock

*COVID-19, coronavirus disease 2019; HFNC, high-flow nasal cannula; ICU, intensive care unit; PaO_2_/FiO_2_, ratio of the partial pressure of oxygen in the arterial blood to the fraction of inspired oxygen; SARS-CoV-2 PCR, severe acute respiratory syndrome coronavirus 2 reverse transcription polymerase chain reaction*.

This study was approved by the Institutional Review Board (IRB) of the Buddhist Taipei Tzu Chi General Hospital (10-X-141) and was conducted in accordance with the principles of the Declaration of Helsinki and its later amendments. The study protocol was reviewed, and an informed consent waiver was received from the IRB. Patient privacy rights, including with regard to any individual person's data in any form (including medical information, images, and videos), were strictly observed.

### Diagnosis and Management

COVID-19 disease severity was defined as either uncomplicated, mild, moderate, severe, or critical illness according to National Institute of Health (NIH) guidelines (8). Uncomplicated COVID-19 involves symptoms such as an upper respiratory tract viral infection and the presence or absence of fever, cough, sore throat, nasal congestion, malaise, headache, and/or muscle pain with no other complications. Mild COVID-19 is defined according to symptoms such as cough with expectoration, fever, general body pain, and weakness. Moderate COVID-19 is associated with a combination of symptoms that are more severe but are not life threatening. Severe COVID-19 describes patients with clinical signs of pneumonia (fever, cough, dyspnea, fast breathing) and one or more of the following symptoms: a respiratory rate of >30 breaths/min, severe respiratory distress, ratio of the partial pressure of oxygen in the arterial blood to the fraction of inspired oxygen (PaO_2_/FiO_2_) of <300, an oxygen saturation (SpO_2_) of <94% on room air, or infiltration of >50%.

In this study and according to the NIH guidelines, patients with critical COVID-19 were confirmed to have a SARS-CoV-2 infection and were admitted to the intensive care unit (ICU) for respiratory and/or cardiovascular organ support, including intubation, rescue strategies, and/or oxygenation (i.e., eliciting changes in the PaO_2_/FiO_2_ ratio). The clinical symptoms characterizing critically severe COVID-19 include the presence of acute respiratory distress syndrome, respiratory failure requiring ventilation, sepsis, and/or septic shock.

The study questionnaire was given to subjects while they were inpatients. The questionnaire included four parts: patient characteristics, type of diet, comorbidities and complications, and pneumonia severity. With regard to patient characteristics, we collected information on sex, age, weight, treatment locations, and diet type (i.e., vegetarian or non-vegetarian). Moreover, we collected data on meat, vegetarian, and other specific diet types retrospectively (i.e., 1 year before the date of COVID-19 diagnosis).

Comorbidities and complications were measured using the Charlson Comorbidity Index (CCI) (9). This index assigns a score for specific diseases, with a higher score indicating a more severe condition and, consequently, a worse prognosis. Underlying comorbidities, such as disabling neurologic conditions, chronic obstructive or restrictive lung disease, coronary atherosclerotic disease, congestive heart failure, liver cirrhosis, end-stage renal disease, diabetes, metastatic cancer, and acquired immunodeficiency syndrome, were recorded.

The severity of pneumonia was measured using the CURB-65 scale (confusion, uremia, respiratory rate, BP, age ≥ 65 years), which is based on confusion (i.e., being newly disoriented with regard to questions relevant to person, place, or time), blood urea nitrogen (BUN) levels of >20 mg/dl, a respiratory rate of ≥30 breaths/min, blood pressure parameters (systolic blood pressure <90 mmHg or diastolic blood pressure ≤60 mmHg), and an age ≥65 years (10). Patients received a score of 1 if any of the symptoms described within the above five items were noted; the highest obtainable score was 5. A score of 0 or 1 indicated that the patient should be treated as an outpatient, a score of 2 indicated a short stay in the hospital or careful observation as an outpatient, and a score of 3–5 indicated that the patient should be hospitalized and that ICU admission should be considered.

### Statistical Analysis

Descriptive statistics were used to present the demographic characteristics of the enrolled study participants. Categorical variables are expressed as numbers and percentages, and continuous variables are expressed as means ± standard deviations.

Categorical variables were examined using the chi-square test and Fisher's exact test, and continuous variables were examined using one-way analysis of variance (ANOVA).

Logistic regression was performed to investigate the factors associated with COVID-19 severity after adjusting for other potentially confounding factors. The data were analyzed using SPSS statistical software (version 24.0, IBM Corp, Armonk, NY, USA). A *p*-value of <0.05 was considered the threshold for statistical significance.

## Results

A total of 906 COVID-19 infected patients comprised the eligible study population (these patients were infected during the year 2021). The study population was enrolled retrospectively during the pandemic to investigate the association between adherence to a vegetarian diet and COVID-19 severity. The hotel of the quarantine center was closed in August 2021. Thus, no more uncomplicated or mild COVID-19 patients were enrolled after August 2021. No patient became critical after the COVID-19 vaccination policy was implemented in our country, so we stopped enrolling patients in August 2021. Therefore, 319 patients who were not admitted to the hospital during the study period (May 2021 to August 2021) were excluded from the current study. A total of 587 patients were examined for eligibility.

**Figure 1:**
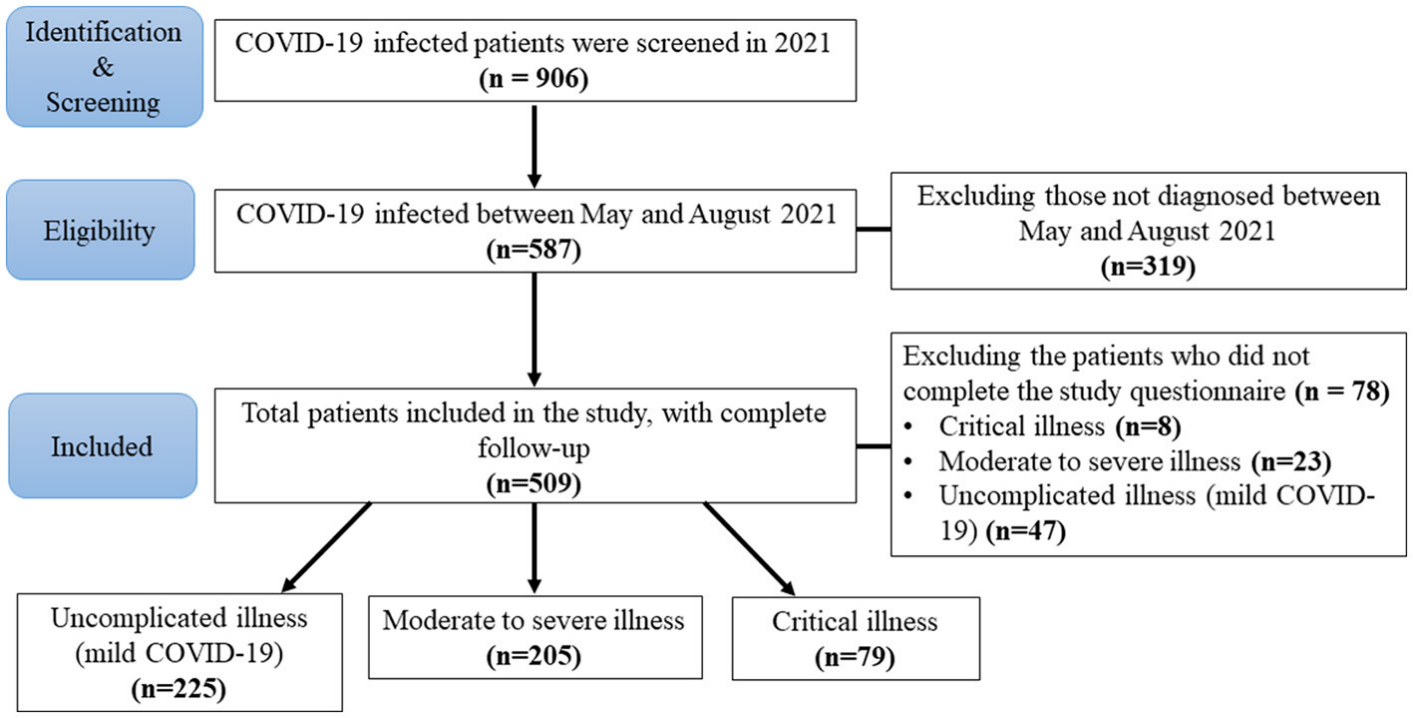
Flow diagram of the study enrollment process. A total of 906 COVID-19 (coronavirus disease 2019) infected patients comprised the eligible study population as of 2021. In order to study vegetarian diets in connection with COVID-19 severity, 319 patients were excluded due to not being evaluated between May 2021 and August 2021. Hence, a total of 587 patients were examined for eligibility. The questionnaire was not completed in detail by 78 patients during admission, which included eight critical, 23 moderate to severe, and 47 uncomplicated (mild) COVID-19 patients. A total of 509 patients with complete follow-up data were included in this study.

The questionnaire was not completed in detail by 78 of these eligible patients during admission, including eight critical, 23 moderate to severe, and 47 uncomplicated COVID-19 cases. Finally, 509 patients were included in this study; these cases had completed the entirety of follow-up (Figure 1).

Table 2 shows subject characteristics as well as the results of statistical analyses comparing patients grouped by severity. Subjects with critical COVID-19 were more likely to be older in age and have a higher body mass index (BMI). A non-vegetarian diet was most prevalent in the critical COVID-19 group, though this finding was not statistically significant.

**Table 2:** Characteristics of COVID-19 patients by severity status.

		**Category**			
	**Total (*N* = 509)**	**Uncomplicated illness to mild COVID-19 (*n* = 225)**	**Moderate to severe COVID-19 (*n* = 205)**	**Critical COVID-19 (*n* = 79)**	***p-*value^†^**
**Sample size**, ***N***					
Sex					0.316
Women	269 (52.8)	119 (52.9)	114 (55.6)	36 (45.6)	
Men	240 (47.2)	106 (47.1)	91 (44.4)	43 (54.4)	
Age, years	52.17 ± 16.57	44.26 ± 14.20	55.66 ± 15.42	65.63 ± 13.85	0.000^*^
Weight (kg)	66.57 ± 13.79	65.97 ± 14.39	66.27 ± 11.89	69.60 ± 16.38	0.169
Body mass index (kg/m^2^)	24.73 ± 4.26	24.16 ± 4.28	24.90 ± 3.52	26.31 ± 5.62	0.002^*^
CCI	1.28 ± 1.35	0.80 ± 1.23	1.48 ± 1.30	2.15 ± 1.28	0.000^*^
CCI = 0	213 (41.8)	137 (60.9)	64 (31.2)	12 (15.2)	0.000^*^
1 ≤ CCI ≤ 2	189 (31.7)	61 (27.1)	96 (46.8)	32 (40.5)	
CCI ≥ 3	107 (26.5)	27 (12.0)	45 (22.0)	35 (44.3)	
Self-reported diets					0.374
Non-vegetarian	487 (95.7)	214 (95.1)	195 (95.1)	78(98.7)	
Vegetarian	22 (4.3)	11 (4.9)	10 (4.9)	1 (1.3)	
Diet type					0.062
Non-vegetarian	487 (95.7)	214 (95.1)	195 (95.1)	78 (98.7)	
Vegan	6 (1.2)	1 (0.4)	5 (2.4)	0 (0.0)	
Ovo-lacto vegetarian	13 (2.6)	10 (4.4)	3 (1.5)	0 (0.0)	
Lacto-vegetarian	1 (0.2)	0 (0.0)	1 (0.5)	0 (0.0)	
Ovo-vegetarian	2 (0.4)	0 (0.0)	1 (0.5)	1 (1.3)	
Pescatarian	0 (0.0)	0 (0.0)	0 (0.0)	0 (0.0)	

† *p-value comparing critical or moderate to severe cases with uncomplicated or mild severity cases. CCI, Charlson Comorbidity Index; COVID-19, coronavirus disease 2019*.

* *p < 0.05*.

In Table 3, we focus on older patients aged ≥65 years. Due to different age groups having very different COVID-19 severities (based on previously published statistics), the older population may be a more indicative group with regard to highlighting the benefits of a vegetarian diet.

**Table 3:** Characteristics of older COVID-19 patients aged ≥65 years by severity status.

		**Category**			
	**Total (*N* = 136)**	**Uncomplicated illness to mild COVID-19 (*n* = 23)**	**Moderate to severe COVID-19 (*n* = 66)**	**Critical COVID-19 (*n* = 47)**	***p*-value^†^**
**Sample size, N**					
Sex					0.316
Women	61 (44.9)	9 (39.1)	34 (51.5)	18 (38.3)	
Men	75 (55.1)	14 (60.9)	32 (48.5)	29 (61.7)	
Age, years	72.46 ± 6.71	69.91 ± 4.51	71.86 ± 6.31	74.55 ± 7.62	0.014^*^
Weight (kg)	65.40 ± 11.21	65.96 ± 14.97	64.30 ± 8.74	66.74 ± 12.06	0.572
Body mass index (kg/m^2^)	24.98 ± 3.65	24.85 ± 4.45	24.82 ± 2.84	25.34 ± 4.28	0.787
CCI	2.94 ± 0.82	3.09 ± 1.12	2.85 ± 0.77	3.00 ± 0.72	0.410
CCI=0	0 (0.0)	0 (0.0)	0 (0.0)	0 (0.0)	
1 ≤CCI ≤2	43 (31.6)	7 (30.4)	24 (36.4)	12 (25.5)	0.471
CCI ≥3	93 (68.4)	16 (69.6)	42 (63.6)	35 (74.5)	
Self-reported diets					0.013^*^
Non-vegetarian	127 (93.4)	19 (82.6)	61 (92.4)	47 (100.0)	
Vegetarian	9 (6.6)	4 (17.4)	5 (7.6)	0 (0.0)	
Particular diet type					0.077
Non-vegetarian	127 (93.4)	19 (82.6)	61 (92.4)	47 (100.0)	
Vegan	3 (2.2)	1 (4.3)	2 (3.0)	0 (0.0)	
Ovo-lacto vegetarian	4 (2.9)	3 (13.0)	1 (1.5)	0 (0.0)	
Lacto-vegetarian	1 (0.7)	0 (0.0)	1 (1.5)	0 (0.0)	
Ovo-vegetarian	1 (0.7)	0 (0.0)	1 (1.5)	0 (0.0)	
Pescatarian	0 (0.0)	0 (0.0)	0 (0.0)	0 (0.0)	

† *p value comparing critical or moderate to severe cases with uncomplicated or mild cases. COVID-19, coronavirus disease 2019*.

* *p < 0.05; crude odds ratio = 19.78; p = 0.009 when evaluating disease severity level in connection with diet type*.

Overall, there were fewer vegetarians in the critical COVID-19 group, indicating a possible protective effect in vegetarians. Moreover, the severity of COVID-19 symptoms showed a borderline significant association (*p* = 0.062) in patients with higher adherence to a vegetarian diet (Table 2). For patients aged ≥65 years, COVID-19 symptom severity was statistically significantly associated (*p* = 0.013) with adherence to a vegetarian diet (Table 3). The crude odds ratio (OR) was 19.78 (*p* = 0.009) when evaluating disease severity level (critical vs. mild disease) in connection with vegetarian or non-vegetarian diet. Subject ages were highest in those with critical COVID-19 (*p* = 0.014).

In further subgroup analysis, the vegetarian group had no differences in BMI (kg/m^2^) when compared to the non-vegetarian group (vegetarian vs. non-vegetarian group: 24.01 ± 3.40 vs. 24.77 ± 4.30, *p* = 0.414). There were no differences between vegetarian and non-vegetarian groups with respect to the other variables, including gender, smoking status, age, weight, CCI, and CURB-65.

We also conducted various subgroup analyses, the results of which are described below. For example, the study population was divided into four subgroups according to age and diet type. We further divided the subjects into low-low risk (age <65 years, vegetarian diet), low-high risk (age <65 years, non-vegetarian diet), high-low risk (age ≥ 65 years, vegetarian diet), and high-high risk (age ≥ 65 years, non-vegetarian diet) subgroups (Supplementary Table 1). In the multivariate analysis, which adjusted for all variables that had a *p* of <0.05 in the univariate analysis (Supplementary Table 1), COVID-19 severity was statistically significantly associated with BMI [adjusted OR = 1.064, 95% confidence interval (CI): 1.017–1.116, *p* = 0.008] and categorization into the “high-high risk” subgroup (adjusted OR = 5.434, 95% CI: 1.624–18.826, *p* = 0.005).

## Discussion

In our study, the severity of COVID-19 symptoms showed borderline significance in patients with higher adherence to a vegetarian diet. Although these findings trend in the direction of a meaningful association between adherence to a vegetarian diet and COVID-19 symptom severity, these findings could also simply be due to chance. Furthermore, when we stratified the study population by groups defined according to age and vegetarian diet status (Supplementary Table 1), COVID-19 severity was found to be statistically significantly associated with older age and a non-vegetarian diet.

Age is well known to be associated with immune system strength and overall immune functioning, and may, thus, reduce individual's capacity to deal with infections effectively and may also affect the likelihood of developing chronic inflammation (11). An age-matched study may help elucidate these findings during future research efforts.

In addition, we evaluated COVID-19 severity in connection with CCI scores, with higher scores associated with greater COVID-19 severity. These results were similar to those of a previous meta-analysis (12), in which each point increase in CCI scores increased mortality risk by 16%. Moreover, a higher mean CCI score was also statistically significantly associated with mortality and disease severity in this prior study. Recently, a well-established observational study likewise explored the association between the CCI and severe COVID-19 outcomes (13). In the current study, we obtained the same results as in previous publications, namely that CCI scores are associated with COVID-19 severity (13).

We note that a recent study suggested strengthening the immune system through dietary habits and specific nutrients (14). However, it is important to acknowledge that dietary habits are changing on many levels worldwide, including as multiple regulations and lockdowns take effect (15, 16).

Prior studies have reported that plant-based diets are nutrient-dense, and include high concentrations of polyphenols, carotenoids, fiber, vitamins A, C, and E, folate, iron, potassium, and magnesium (17, 18). Plant-based diets have known benefits in terms of preventing hypertension and cardiovascular disease (19, 20). Vegetarian plant-based diets also strengthen the immune system, reduce inflammation and oxidative stress (14), and may help prevent chronic kidney disease and preserve kidney function (21). Hence, we propose the hypothesis that vegetarian diets might prevent heart and kidney diseases as well as strengthen the immune system and lower comorbidities. The presence of fewer comorbidities may influence the severity of COVID-19 indirectly.

An epidemiologic prospective cohort study by Merino et al. (1) suggests that plant-based foods were associated with lower severity of COVID-19, especially in areas of higher socioeconomic deprivation. In our study, the hospital setting enrolled a more severe and critical COVID-19 population and when there was no COVID-19 vaccination during pandemics, our findings agree with the results of their study. However, we did not explore other supplements of Vitamin B12, D3, or blood tests in our study population because it was a retrospective study. Therefore, we recommend that future studies include vaccination status and vitamin supplements intake data to clarify the components of a vegetarian diet that may reduce COVID-19 severity.

This study has several limitations. First, too few vegetarians were recruited in this study, thus limiting our study power. Moreover, our study population did not include any patients with asymptomatic COVID-19 and our study population may thus be limited in terms of representativeness, generalizability, and external validity. Further, large study populations and multi-center studies in quarantine hotels and responding hospitals are needed to further clarify the possible protective effect on vegetarians of old age.

Second, the study questionnaire was not sufficiently detailed. For example, we only asked about the adherence to a vegetarian diet over the course of 1 year. A more detailed and long-term investigation of vegetarian as compared with non-vegetarian diets is necessary in order to clarify the relationship between diet and COVID-19 severity in the future.

## Conclusion

For older COVID-19 patients, we found that a non-vegetarian diet was associated with a higher risk of critical COVID-19 severity. Additional research is necessary in order to determine the effects of dietary habits on COVID-19 risk and severity during the global pandemic.

## Data Availability Statement

The raw data supporting the conclusions of this article will be made available by the authors, without undue reservation.

## Ethics Statement

For this retrospective study, informed consent was waived by the IRB, and the privacy rights of patients, which cover any individual's data in any form (including individual details, images, or videos), were upheld. This study was approved by the Institutional Review Board of Taipei Tzu Chi Hospital, Buddhist Tzu Chi Medical Foundation (approval number 10-X-141) and conducted according to the amended Declaration of Helsinki. Written informed consent for participation was not required for this study in accordance with the national legislation and the institutional requirements.

## Author Contributions

Y-CH and Y-CC conceived the study, designed the trial, obtained the research funding, analyzed and interpreted the data, and contributed to manuscript preparation. Y-CH and W-LS supervised the conduct of the trial, as well as data collection, and revised the manuscript for critical content. W-LS provided statistical advice with regard to the study design, and analyzed the data. Y-CH drafted the manuscript, and all authors contributed substantially to its revision. All authors take responsibility for the article as a whole. All authors contributed to the article and approved the submitted version.

## Funding

This study was supported by a grant from the Taipei Tzu Chi Hospital, Buddhist Tzu Chi Medical Foundation (TCRD-TPE-109-RT-10). The funder had no role in the study design, data collection and analysis, decision to publish, or preparation of the manuscript.

## Conflict of Interest

The authors declare that the research was conducted in the absence of any commercial or financial relationships that could be construed as a potential conflict of interest.

## Publisher's Note

All claims expressed in this article are solely those of the authors and do not necessarily represent those of their affiliated organizations, or those of the publisher, the editors and the reviewers. Any product that may be evaluated in this article, or claim that may be made by its manufacturer, is not guaranteed or endorsed by the publisher.
