# Supplementary material for: COVID-19 Illness Severity in the Elderly in Relation to Vegetarian and Non-vegetarian Diets: A Single-Center Experience
Source: Front Nutr. 2022 Apr 29;9:837458. doi: 10.3389/fnut.2022.837458 (PMC9101048; doi:10.3389/fnut.2022.837458)
Supplement: Supplementary file 1 [file Table_1.docx]

| Table S1. Logistic regression results, univariate and adjusting for potential confounders. | | | | |
| --- | --- | --- | --- | --- |
|  | Univariate | | Multivariate | |
|  | OR (95% CI) | p-value | Adjusted OR (95% CI) | p-value |
| Subgroup |  |  |  |  |
| Low-low | Ref |  | Ref |  |
| Low-high | 0.878 (0.286–2.780) | 0.819 | 0.865 (0.280–2.752) | 0.799 |
| High-low | 1.458 (0.263–8.506) | 0.665 | 1.580 (0.282–9.296) | 0.601 |
| High-high | 5.403 (1.624–18.603) | 0.006* | 5.434 (1.624–18.826) | 0.005* |
| Body mass index | 1.065 (1.019–1.114) | 0.006* | 1.064 (1.017–1.116) | 0.008* |
| Gender | 1.088 (0.757–1.565) | 0.649 | 0.834 (0.560–1.238) | 0.370 |

Dependent variable: COVID-19 severity

OR, odds ratio; CI, confidence interval; *Statistically significant (p <0.05)
